# Large-scale SARS-CoV-2 sequencing indicates prior community circulation of the viral strain associated with Germany’s largest meat processing plant

**DOI:** 10.1038/s41598-025-25764-0

**Published:** 2025-11-04

**Authors:** Jessica Nicolai, Abigail J. Miller, Naja Hahn, Julia Fazaal, Anne Bunte, Janine Silvery, Marian Rosenstengel, Per Hoffmann, Kerstin U. Ludwig, Carsten Tiemann, Laura E. Rose, Alexander T. Dilthey

**Affiliations:** 1 Institute of Population Genetics, Heinrich-Heine University Düsseldorf, Düsseldorf, Germany; 2 Institute of Medical Microbiology and Hospital Hygiene, University Hospital Düsseldorf, Heinrich Heine University Düsseldorf, Düsseldorf, Germany; 3 Institute of Human Genetics, School of Medicine, University Bonn & University Hospital Bonn, Bonn, Germany; 4 Public Health Authority of Gütersloh District, Gütersloh, DE Germany; 5 MVZ Labor Krone eGbR, Bad Salzuflen, Germany; 6 Center for Digital Medicine, Heinrich Heine University Düsseldorf, Düsseldorf, Germany

**Keywords:** Public health, Pathogens, Virology, SARS-CoV-2

## Abstract

A SARS-CoV-2 outbreak at a meat processing plant (MPP) in the German district of Gütersloh accounted for 18% of Germany’s SARS-CoV-2 cases in June 2020 and was subject of intense public interest, including the speculation that the outbreak strain may have been imported by foreign MPP workers. We sequenced the SARS-CoV-2 genomes of 1,438 SARS-CoV-2 samples collected from Gütersloh MPP workers for serial diagnostic testing and screening purposes (“outbreak samples”; approximate case coverage 68%) and of 157 samples collected from Gütersloh-area cases for routine diagnostic purposes (“community samples”). Greater than 98% of outbreak samples carried the outbreak-associated strain, defined by eight mutations and lineage B.1.329, confirming the overall clonality of the outbreak and showing that potential secondary introductions of other viral lineages had an at most limited role. Of fifteen viral sub-lineages detected in early outbreak-associated samples sequenced by another study, only one showed substantial persistence into the peak outbreak period, suggesting that transmission dynamics within the MPP were influenced by bottlenecks and superspreading-like patterns. While the detection of B.1.329 in community samples peaked during the outbreak, it was found to be present in community samples between March and September 2020, with the first exact matches to the outbreak strain appearing in April 2020. We found no epidemiological connections between early B.1.329-carrying community cases and the MPP, and a GISAID search for B.1.329 did not identify any samples collected outside of Germany. The outbreak strain was therefore likely circulating within the community before the outbreak and there was no indication of importation by MPP workers. Our study demonstrates how large-scale viral genome sequencing can contribute to the investigation of outbreaks and inform public discourse.

**Supplementary Information:**

The online version contains supplementary material available at 10.1038/s41598-025-25764-0.

## Introduction

Understanding pathogen transmission is key to the implementation of effective infection prevention measures. During the initial phase of the COVID-19 pandemic, “superspreading”-type transmission played an important role^1–5^; in addition to high susceptibility to SARS-CoV-2 in immunologically naïve populations, superspreading was enabled by an incomplete understanding of the underlying epidemiological and behavioral factors associated with superspreading. Superspreading events also played an important role in public discourse during the pandemic, often in the context of discussions around infection prevention measures and their responsible implementation—or failure thereof—by individuals or corporations^6,7^.

In June 2020, a large SARS-CoV-2 outbreak was detected at Germany’s largest meat processing plant (MPP) in the district of Gütersloh. The outbreak involved at least 2,119 cases and accounted for 18% of all German SARS-CoV-2 cases in June 2020^8,9^; while the number of outbreak cases peaked in the second half of June, it was later established that the outbreak strain had been present in the Gütersloh MPP since the second half of May 2020^10^. In response to the outbreak and to prevent transmission into the local community, the state government of North-Rhine Westphalia issued a lockdown for the district of Gütersloh, starting on 23 June, which included contact restrictions as well as the closure of leisure facilities such as museums and swimming pools^11^. The outbreak and the potential origins of the outbreak-associated viral strain were matters of intense public interest, with transmission during a church service attended by multiple workers of the Gütersloh MPP speculated to have played a role^12,13^. It was also speculated that foreign workers of the MPP Gütersloh may have “imported” the outbreak-associated viral strain into Germany when returning from holidays in their home countries; this theory was put forward by political leaders as well as by representatives of the company operating the MPP in Gütersloh^14,15^.

A comprehensive analysis of the Gütersloh outbreak by Günther et al.^10^ based on viral genome sequencing and epidemiological information provided by the company operating the Gütersloh MPP led to the following main conclusions. First, the outbreak was clonal, and the outbreak-associated viral strain had been present in the population of Gütersloh MPP workers for approximately four weeks prior to the rapid accumulation of outbreak cases in mid-June 2020. Second, the introduction of the viral strain into the population of Gütersloh MPP workers could be traced back to a contact on 17 May 2020 between two Gütersloh MPP workers and two workers from an MPP in the nearby town of Dissen. The latter facility, operated by a different company, was also affected by a SARS-CoV-2 outbreak in mid-May 2020. Third, superspreading from one of the two Gütersloh MPP workers to colleagues from the same shift in the period between 18 and 20 May 2020 likely played an important role in the spread of the virus in the Gütersloh MPP worker population. Fourth, systems and various environmental and working condition-related factors contributed to creating an environment that permitted the rapid spread of SARS-CoV-2 in the Gütersloh MPP during the superspreading event and thereafter. The investigation of Günther et al. included the analysis of 37 SARS-CoV-2 genomes; 20 of these were obtained from Gütersloh MPP cases registered in May 2020 (“early Gütersloh cases”); 15, from Gütersloh MPP cases registered in June 2020 (i.e. during the later phase of the outbreak); and, finally, 2, from the two workers of the other MPP involved in the initial transmission event. The findings of Günther et al. are in line with the international literature on SARS-CoV-2 outbreaks in food processing facilities; during the COVID-19 pandemic, numerous outbreaks of SARS-CoV-2 in such facilities were documented globally^16,17^ and it is well-established that the working conditions in food and meat processing plants, such as low temperatures and very high or low relative humidities, are conducive to the rapid spread of SARS-CoV-2^18^, rendering such facilities potential hotspots for SARS-CoV-2 transmission.

Here we report on an extended investigation of the Gütersloh SARS-CoV-2 outbreak that is based on large-scale genome sequencing. Compared to the study of Günther et al.^10^, the set of SARS-CoV-2 genomes analyzed by us is more than 40 times larger, achieving a much higher coverage of outbreak cases and also including SARS-CoV-2 cases from the local community. We complement and extend the analysis of Günther et al.^10^ by specifically addressing the following questions: First, can we confirm the clonal nature of the outbreak as reported by Günther et al., or is there evidence for the presence of additional low-frequency independent introductions? Second, are the collected genome sequence data informative about prior community circulation of the strain in the Gütersloh area or importation from another country? Third, did the outbreak spread from the MPP to the local community and how long did it persist? Fourth, which of the viral sub-lineages present in the set of 20 SARS-CoV-2 genomes from “early” Gütersloh MPP cases (registered in May) in the Günther et al. analysis persisted into the main flare-up phase of the outbreak in June 2020?

## Material and methods

### SARS-CoV-2 sample collection and sequencing

SARS-CoV-2 genome sequencing was retrospectively carried out from positive diagnostic swabs obtained from Gütersloh MPP workers for serial diagnostic testing and screening purposes (“outbreak samples”, mainly collected on site), as well as from diagnostic swabs from Gütersloh-area cases sent to Labor Krone, a large diagnostic laboratory in the German region of East-Westphalia, for routine diagnostic testing purposes (“community samples”). The generation of viral genome sequencing data from routine diagnostic samples for outbreak investigation purposes (i.e. retrospectively) was commissioned by the Ministerium für Arbeit, Gesundheit und Soziales des Landes Nordrhein-Westfalen (Ministry for Work, Health and Social Affairs of the State of North Rhine-Westphalia), the highest public health authority of the state of North-Rhine Westphalia. The study design was retrospective in nature. The study was carried out with waived informed consent in compliance with the Institutional Review Board of the Medical Faculty of Heinrich Heine University. All methods were performed in accordance with the relevant guidelines and regulations. Of note, our study did not involve any re-sequencing of samples collected by Günther et al.

Viral RNA was extracted using the NucleoMag VET kit (Machery-Nagel, Germany) based on a paramagnetic particle technology on the KingFisher system (ThermoFisher, Germany). SARS-CoV-2 was detected by real-time PCR using the Allplex 2019-nCoV assay (Seegene, Germany) and the CFX96 Real-time PCR System (Biorad, Germany). The reagents and protocols were used in accordance with the manufacturer’s instructions. SARS-CoV-2 sequencing was carried on the Illumina NovaSeq 6000 platform, employing the COVIDSeq Test protocol. The sample collection dates of six MPP worker samples (9,003,200,592, 9,003,200,624, 1,055,341,365, 9,003,205,231, 1,042,921,582, 1,042,921,583) with implausible sample collection dates (16 March 2020, 25 March 2020, 21 September 2020) were set to “NA”.

### Viral genome assembly and quality control

Paired-end Illumina sequencing data for each sample generated with CovidSeq were aligned with BWA-MEM 0.7.15^19^ against a combined reference that consisted of i) the SARS-CoV-2 reference genome; ii) the GRCh38 human reference genome; iii) and 105 other viral reference genomes to capture reads generated by potential non-specific amplification. BAM files were generated with samtools 1.6^20^. A viral consensus sequence for each sample was generated using iVar 1.0^21^ (commit 6c0d75867204be2b3f2e54f58b40cb411ddd1505), based on the command “samtools mpileup -aa -A -d 0 -B -Q 0 -r SARS-CoV-2 –reference $refGenome $BAM | ivar consensus -p $outputPrefix -t 0.5 -m 20”, where $refGenome pointed to the combined reference file; $BAM, to the BAM of the sample; and $outputPrefix, to the output filename prefix for the sample. The number of undefined characters (“N”) was determined for all sequenced genomes and all genomes with more than 3000 undefined characters were excluded from further analyses.

### Lineage, clade and variant analyses

Pangolin^22^ was used to determine the lineage of the sequenced SARS-CoV-2 genomes. Nextclade^23^ was used for clade assignment and to determine the substitutions present in the sequenced viral genomes relative to the reference genome from Wuhan^24^ (MN908947.3).

### Günther et al. sub-lineage analysis

Unique viral haplotypes in the set of 35 SARS-CoV-2 genomes sequenced by Günther et al.^10^ were enumerated and named after the ID of the first sample carrying the corresponding haplotype in the analysis of Günther et al.; for this analysis, a haplotype was defined by the pattern of detected mutations compared to the SARS-CoV-2 reference genome (see Supplementary Fig. 1). For each haplotype, we counted the number of exact matches (i.e., containing the same set of mutations relative to the SARS-CoV-2 reference genome) in our set of sequenced SARS-CoV-2 genomes.

### Analysis of epidemiological connections between B.1.329-carrying community cases and the Gütersloh MPP

To investigate whether the strain associated with the Gütersloh MPP outbreak was in prior community circulation in the Gütersloh area, we searched the dataset of sequenced SARS-CoV-2 samples collected from Gütersloh-area cases for routine diagnostic purposes for the outbreak-associated B.1.329 lineage of SARS-CoV-2 and registered before 15 June 2020. For each identified case, we carried out an in-depth analysis of the identified mutations compared to the outbreak strain. To determine whether the identified cases were epidemiologically connected to the Gütersloh MPP, we evaluated case and contact tracing data collected by Gütersloh Health Authority, based on the classification of COVID-19 as a notifiable disease under the German *Infektionsschutzgesetz* (German Infection Protection Act), for potential links to the MPP and the population of MPP workers. The reviewed data included the names, addresses, occupations and routine contact tracing data. Contact tracing was carried out for all registered non-outbreak cases in the Gütersloh district during the year of 2020. Participation in contact tracing interviews was mandatory. All personally identifiable information remained at the Gütersloh Health Authority. To assess potential transmission from the outbreak into the local community, we counted the number of community cases registered during or after the outbreak which were assigned to the B.1.329 lineage.

### Use of generative AI

This manuscript was created using ChatGPT for AI-assisted copyediting purposes.

## Results

### Sequencing of SARS-CoV-2 MPP outbreak and community samples

To characterize the genetic structure of the Gütersloh outbreak, we attempted to sequence SARS-CoV-2 from 2,241 archived SARS-CoV-2 positive diagnostic swab samples; 1,822 of these were collected from Gütersloh MPP workers for serial diagnostic testing and screening purposes (“outbreak samples”) and 419 collected from Gütersloh-area cases for routine diagnostic testing by Labor Krone (“community samples”). The sample collection dates of the MPP worker samples ranged from 05 June to 23 July 2020, with the large majority (91%) of samples collected during the peak period of the outbreak from mid- to late June. For community samples, sample collection dates ranged from 11 March to 10 October 2020. Of note, the definition of community samples as collected independently of serial screening and testing efforts in the MPP did not preclude the presence of epidemiological connections between the corresponding cases and the MPP, which we therefore investigated separately based on Gütersloh Health Authority data where relevant to our key conclusions (see sections below).

Sequencing was deemed successful if the reconstructed viral genome had ≤ 3000 undefined characters (“Ns”). The final set of analyzed sequences comprised 1,595 viral genomes, of which 1,438 genomes were from outbreak samples. This corresponded to an approximate outbreak case coverage of 68% (calculated as 1,438 / 2,119); the other 157 genomes were from community samples.

### Clonality of the Gütersloh MPP outbreak

**Fig. 1:**
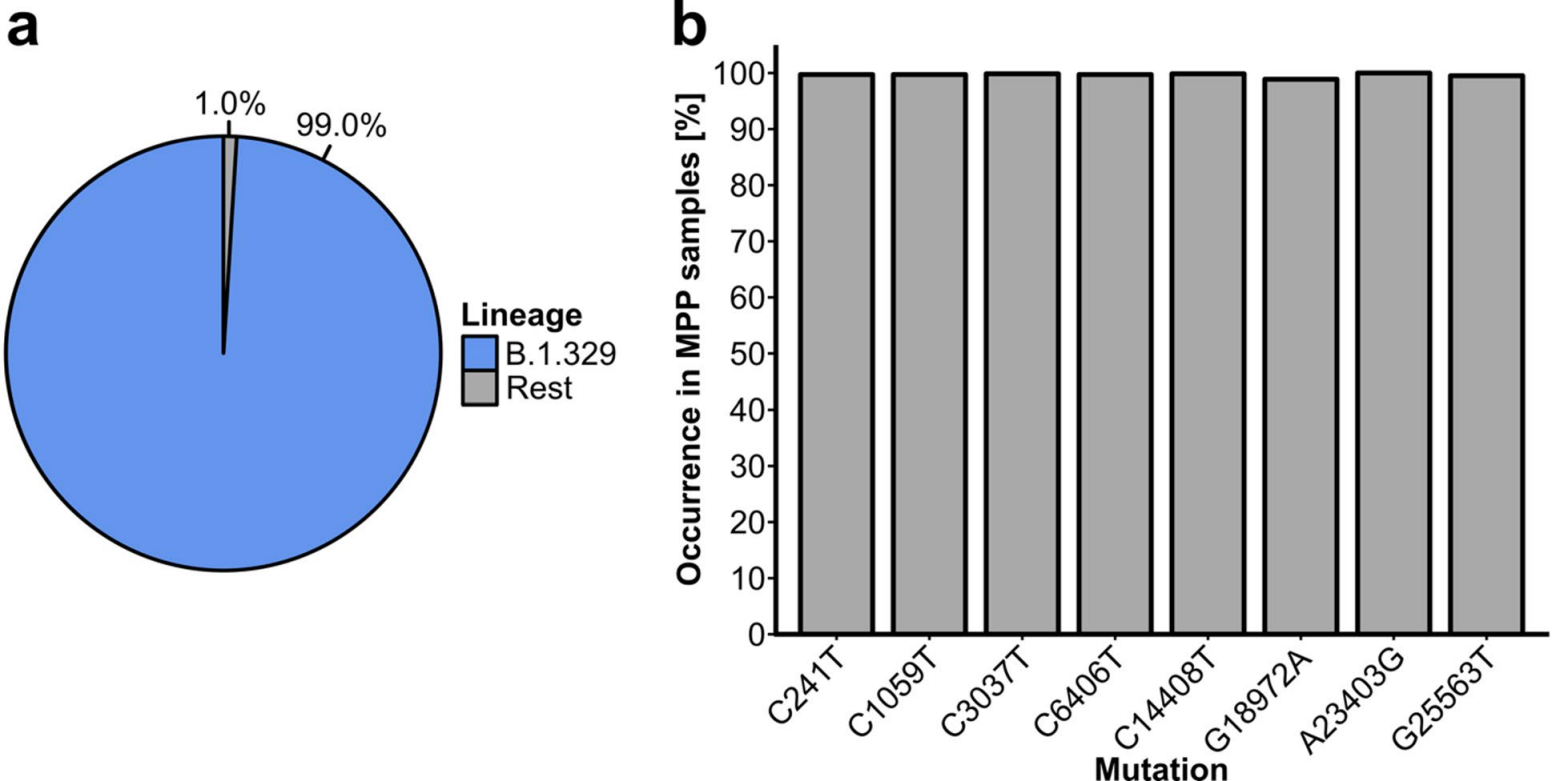
Presence of B.1.329 and outbreak-defining substitutions in sequenced outbreak samples. (**a**) Lineage assignment of the sequenced outbreak samples. (**b**) Frequency of the outbreak-defining substitutions in the sequenced outbreak samples.

We compared the sequenced outbreak samples to the reference genome from Wuhan^24^ (MN908947.3) using Nextclade^23^. Across all outbreak samples, we detected 529 unique substitutions (mean number of mutations per sample = 8.9 +/− 1.1). Eight substitutions (C241T, C1059T, C2027T, C6406T, C14408T, G18972A, A23403G and G25563T) were present in at least 98.9% and up to 100% (A23403G) of outbreak samples (Fig. 1B). Furthermore, all eight substitutions were present in 98.2% (n = 1412) of outbreak samples. Nearly 42% of outbreak samples (n = 602) contained these eight and no other substitutions. We thus declared these substitutions as the “outbreak-defining substitutions”. Furthermore, 1,424 outbreak samples (99.0%) were assigned to lineage B.1.329, while the remaining 14 (1.0%) were assigned to three other lineages; we therefore declared B.1.329 as the outbreak lineage (Fig. 1A). The analysis of individual mutation patterns and the lineage-level analysis thus indicated that the outbreak was clonal. Moreover, the frequency of cases potentially associated with other viral lineages remained below 2%, indicating an at most minor contribution of secondary introduction events to the overall case load of the outbreak.

### Presence of B.1.329 in sequenced community samples

**Fig. 2:**
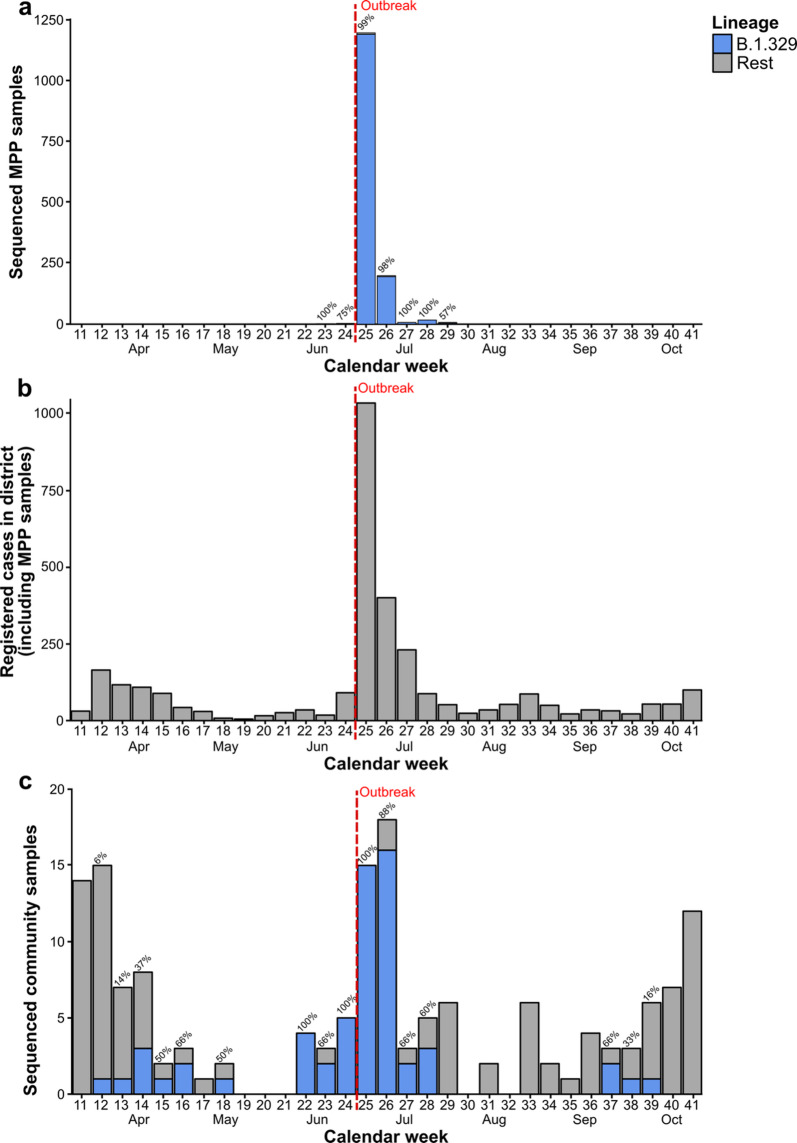
Weekly case numbers in the MPP and the surrounding district. (**A**) Sequenced outbreak samples stratified by lineage and calendar week. (**B**) Registered cases in Gütersloh district by calendar week (including outbreak cases). (**C**) Sequenced community samples by lineage and calendar week. Percentages above the bars indicate the proportion of sequenced cases assigned to the outbreak lineage B.1.329, if applicable. Community samples were defined as samples collected from Gütersloh-area cases for routine diagnostic testing purposes.

We investigated the frequency of the outbreak-associated lineage B.1.329 in sequenced community samples, i.e. in samples collected from Gütersloh-area cases for routine diagnostic testing purposes (Fig. 2). B.1.329 was first detected in a community sample collected in calendar week 12 of 2020, i.e. 14 weeks prior to the peak of the MPP outbreak in calendar week 26. The B.1.329 strain circulated in the community until at least calendar week 18, followed by a three-week period (weeks 19–21) without any sequenced community samples. Between calendar weeks 22 and 28, corresponding to the period a few days after the occurrence of superspreading in the Gütersloh MPP to approximately two weeks after the peak of the outbreak (25 May–12 July), B.1.329 accounted for 60% to 100% of the sequenced community samples. Afterwards, B.1.329 was not detected in the community for an 8-week period, until it briefly re-appeared at low absolute sample counts over a 3-week period in the fall.

The detection of B.1.329 in terms of the absolute numbers of B.1.329-carrying sequenced community cases generally mirrored the short-term outbreak-associated increase in overall SARS-CoV-2 cases in Gütersloh between calendar weeks 24 and 28, while also showing community circulation in the periods before and after the outbreak. Outside of the outbreak period, SARS-CoV-2 case numbers in Gütersloh generally followed the development of case numbers in Germany over the course of 2020^25^, exhibiting a spike in case numbers in the spring, a decrease during the summer, and another period of increase in the fall (Fig. 2A). Similarly, the clade distribution of sequenced community samples collected outside of the outbreak period was similar to that observed across Germany (Supplementary Fig. 2). In terms of Pango lineages, we detected a total of 9 different lineages (including the outbreak lineage B.1.329), most of which were sub-lineages of B.1, in the pre-outbreak period; in the post-outbreak period, we detected a total of 21 lineages, including the outbreak lineage (Supplementary Table 1).

### Prior circulation of the outbreak-associated strain in the community, independent of the MPP

**Fig. 3:**
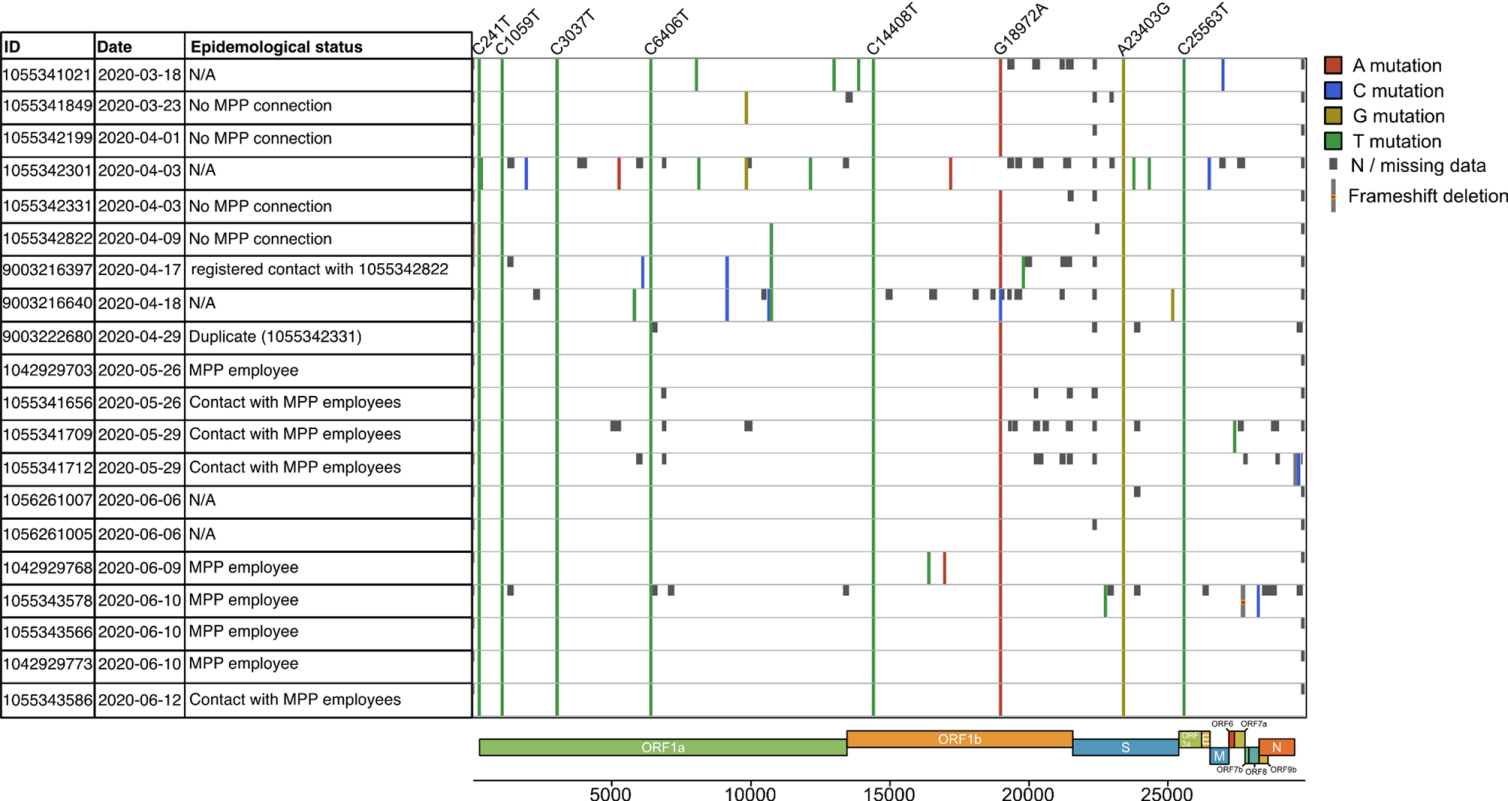
B.1.329 community samples collected prior to the outbreak. The first three columns show sample ID, sample collection date, and whether case and contact tracing data indicated any epidemiological links between the sample and the MPP. The samples were compared to the Wuhan reference genome using Nextclade. Colored lines indicate SNPs compared to the reference genome, the color indicating the detected variant allele differing from the reference. The eight outbreak-defining mutations are listed on the top. Missing data is represented by dark gray boxes. Light gray lines with a red square indicate a frameshift deletion. Parts of this figure are based on the result output from Nextclade.org^23^.

To investigate the potential community circulation of the outbreak-associated strain in the pre-outbreak period, we first carried out a fine-scale mutational analysis of the 20 B.1.329-carrying community samples collected prior to the peak of the outbreak in June 2020 (Fig. 3). Nine B.1.329-carrying samples were collected before May 2020 (calendar week 18); of these, three carried all eight outbreak-defining substitutions and no others. Furthermore, based on case and contact tracing data collected by Gütersloh Health Authority, we investigated 15 of the 20 identified B.1.329 community samples for potential epidemiological connections between the cases that these samples were collected from and the MPP (Fig. 3); of note, this set included all identified B.1.329-carrying samples with collection dates before May 2020. For 11 of the 15 samples investigated, we identified an epidemiological connection between the corresponding cases and the MPP; the earliest sample collection date in this set was 26 May. For the remaining four samples, no epidemiological connections between the corresponding cases and the MPP were detected; two of these, collected in early April (Fig. 3; sample IDs 1,055,342,199 and 1,055,342,331), carried exactly the set of eight outbreak-defining mutations. In conclusion, we detected the outbreak-associated strain in community samples without any apparent connections to the MPP more than six weeks prior to the initial transmission of the outbreak strain to Gütersloh MPP workers.

### No indication of the presence of B.1.329 in other countries

We carried out a GISAID^26^ search for B.1.329 and identified 1,686 samples (Supplementary Table 2); 1,626 of these were submitted by us as part of this project. All identified samples were collected in 2020 and none of the identified samples were collected in countries other than Germany (Supplementary Fig. 3; see Supplementary Table 2 for details). Of the 60 B.1.329 samples found in GISAID that were not part of this study, 35 were uploaded by Günther et al.; the remaining 25 samples were uploaded by the University of Bielefeld, had sample collection dates between 08 June 2020 to 20 June 2020, and were collected in the city of Bielefeld, also located in the German state of North-Rhine Westphalia, approximately 20 km from Gütersloh. Based on this information, the earliest detection of B.1.329 was in the community samples sequenced as part of this study.

### Limited evidence for the persistence of multiple viral sub-lineages in the peak period of the outbreak

To determine the extent to which the fifteen viral sub-lineages (“haplotypes”; see Methods and Supplementary Fig. 1) present among the 35 SARS-CoV-2 genomes sequenced by Günther et al.^10^ persisted into the peak period of the outbreak, we searched our set of MPP samples for exact matches to these. For five sub-lineages, named after the ID of the first samples carrying the corresponding haplotype in Günther et al., at least one exact match was detected. For sub-lineage B1, defined by the set of 8 outbreak-defining mutations, 602 exact matches in our MPP dataset were detected; the corresponding sample B1 was collected from one of the two MPP workers involved in the initial transmission of the outbreak strain into the population of Gütersloh MPP workers. For sub-lineages O9, O7, and O2, we detected 23, four, and three matches, respectively; the corresponding samples were collected in mid-June and thus fell into the period during which the majority of our MPP samples were collected. Finally, we detected one exact match to sub-lineage B2; the corresponding sample was collected from the other MPP worker involved in the initial transmission of the outbreak strain into the population of Gütersloh MPP workers. In conclusion, of the sub-lineages present in samples collected during the early outbreak period in May 2020 in the Günther et al. analysis, apart from a single match to B2, only B1 was found to have persisted into the peak period of the outbreak.

## Discussion

We carried out a comprehensive investigation of the Gütersloh MPP outbreak based on large-scale genome sequencing. Compared to an earlier analysis by Günther et al.^10^, our analysis was based on a much larger set of sequenced viral genomes (1,595 v/s 35); achieved a much higher SARS-CoV-2 sequencing rate of outbreak-associated cases (~ 68%), including cases registered during the peak period of the outbreak in June 2020; and involved the analysis of community samples. To the best of our knowledge, our dataset is the largest existing outbreak sequencing dataset in terms of the number of sequenced cases.

Our analysis of the generated sequencing data led to the following results. First, the outbreak-associated viral strain, defined in terms of outbreak-defining mutations or by the Pango lineage B.1.329, accounted for at least 98% of sequenced outbreak cases; conversely, the frequency of other viral lineages remained below 2%. Second, B.1.329 was found in community samples, defined as samples collected from Gütersloh-area cases for routine diagnostic testing purposes, between March and September 2020; outside of the outbreak period, the absolute number of B.1.329-carrying community samples was low but spiked during the outbreak period (due to how community samples were defined, however, the frequency of B.1.329 in the sequenced community samples should not be interpreted as an estimate of its frequency in the general Gütersloh area population; see “Limitations”). Third, the outbreak strain, defined by the presence of the eight outbreak-defining mutations and no others, had been present in community samples since early April, i.e. approximately six weeks prior to the Gütersloh MPP outbreak. Furthermore, we did not detect any epidemiological links between the early community cases carrying the outbreak strain and the MPP. Fourth, based on a GISAID search, the presence of B.1.329 was not detected outside of the German state of North-Rhine Westphalia (the carrying of the outbreak strain by two workers of the Dissen MPP, as reported by Günther et al., however, indicated the presence of B.1.329 in the German state of Lower Saxony). Fifth, of the fifteen viral sub-lineages present in Günther et al.’s set of sequenced samples from May 2020, only B1 – carrying the outbreak-defining mutations and no others – had substantially persisted into the peak period of the outbreak.

What are the implications of these findings with respect to viral transmission within the Gütersloh MPP outbreak? First, the Gütersloh MPP outbreak was clonal over the complete outbreak-associated period, including the peak period of the outbreak in June 2020. The contribution of secondary introduction events of other viral lineages to the overall case load of the outbreak, if these happened at all, remained below 2%. Of note, as the outbreak-associated lineage B.1.329 was also found to be in community samples, secondary introduction events of the same viral lineage could not be excluded. While we did not attempt to characterize individual transmission chains within the MPP in detail, the limited persistence of early sub-lineages, associated with the three-day superspreading period in the Gütersloh MPP in late May that was described by Günther et al., into the peak period of the outbreak suggested that the ongoing transmission dynamics within the MPP were influenced by bottlenecks and superspreading-like patterns. Of note, the sharp decline of registered outbreak cases in calendar weeks 26 and 27 (Fig. 2) indicated that the outbreak management measures enacted by Gütersloh Health Authority were successful and that ongoing viral transmission in the Gütersloh MPP was suppressed.

Was the outbreak strain transmitted from the MPP to the local community, consistent with an importation of the strain by MPP workers, or was the strain in prior community circulation? While it is not possible to establish the definitive direction of transmission from genome sequencing data, the detection of the outbreak-associated strain in the local community six weeks prior to the Gütersloh MPP outbreak in cases not connected to the MPP, as well as the lack of detection of B.1.329 in any other countries, suggested that the outbreak-associated viral strain was in prior community circulation and therefore not imported by MPP workers. In this context, it is important to note that B.1.329 was likely already transmitting in the population of Dissen MPP workers before it spread to the Gütersloh MPP; Günther et al. sequenced the viral genomes of the two Dissen MPP workers involved in the transmission event on 17 May 2020 and found that they carried B.1.329. As was the case for the Gütersloh MPP, B.1.329 may already have been present in the worker population of the Dissen MPP workers for two or three weeks before its ongoing transmission in the plant was detected through serial testing in mid-May. Even accounting for the potential presence of B.1.329 in Dissen MPP from late March, however, its presence in the community of Gütersloh can still be assumed to precede its presence in the population of Dissen MPP workers by at least three weeks, thus rendering transmission from the community to Dissen MPP more likely than transmission from Dissen MPP to the community.

It is difficult to know which impact the availability of knowledge—which could, for example, been generated in a timely manner using large-scale real-time SARS-CoV-2 genome sequencing^27,28^—of the outbreak’s genetic structure and of the likely community origin of the outbreak-associated viral strain would have had at the time of the outbreak. With respect to the response to the outbreak, it seems plausible that a more immediate recognition of the clonal outbreak situation in the plants and of the links between the two outbreaks could have contributed to occupational health and safety measures and an even earlier implementation of forceful containment measures. In the aftermath of the outbreak, viral genome sequencing was rapidly established as a tool widely used by Gütersloh Health Authority and other local public health authorities in the German region of East-Westphalia for the investigation of the SARS-CoV-2 outbreaks. With respect to the public discourse surrounding the outbreak, recognition of the prior community circulation of the outbreak-associated viral strain would likely have defused the politically charged allegation that the outbreak-associated viral strain may have been imported by non-German workers of the MPP. Our study therefore gives an example of how genome sequencing can not only inform the investigation of pathogen transmission in large outbreaks, but also the public discourse that often surrounds these.

Limitations: As our study did not determine the ultimate index case of the outbreak-associated strain in the population, an importation event cannot be completely ruled out. Systematic genomic surveillance of SARS-CoV-2 in Germany was only implemented beginning in late 2020 when the “Coronavirus-Surveillanceverordnung” was enacted. The fact that GISAID did not contain any B.1.329 samples from countries other than Germany does not rule out its presence in other countries, in particular not in countries with lower SARS-CoV-2 sequencing activity; Brito et al.^29^ analyzed the weekly sequenced cases per country and found that between March 2020 and February 2022 only 13 out of 189 countries sequenced a high enough percentage of cases (> = 5%) to reliably detect rarer viral lineages with a prevalence of 0.1–1.0%. Bulgaria and Romania, countries of origin for many Gütersloh MPP workers, did not belong to these, with only 132 and 15 sequences with sample collection dates prior to 01 June 2020 uploaded to GISAID from Romania and Bulgaria, respectively^26^. Furthermore, community samples were defined as samples collected from Gütersloh-area cases for routine diagnostic testing purposes; this definition does not rule out epidemiological connections between the cases that these samples were connected from and the MPP, and our analysis of B.1.329-carrying samples with collection dates before June 2020 showed that such links sometimes exist. The frequency of B.1.329 in the sequenced community samples should therefore not be interpreted as an estimate of the frequency of B.1.329 in the general population of Gütersloh area. With respect to B.1.329 cases in the community detected in the post-outbreak period, these may represent ongoing community transmission or transmission chains that originated from the MPP outbreak. Finally, we did not attempt to characterize the global origins of the outbreak lineage B.1.329, and we did not attempt to characterize fine-scale transmission patterns within the MPP.

## Supplementary Information

Below is the link to the electronic supplementary material.

Supplementary Material 1

Supplementary Material 2

Supplementary Material 3

Supplementary Material 4

Supplementary Material 5

## Data Availability

The analyzed viral consensus sequences are available on GISAID ([https://gisaid.org/] (https:/gisaid.org); see Supplementary Table 3 for accession numbers) and provided as part of the Supplement (Supplementary File 1).
