# Supplementary material for: Large-scale SARS-CoV-2 sequencing indicates prior community circulation of the viral strain associated with Germany’s largest meat processing plant
Source: Sci Rep. 2025 Nov 4;15:38466. doi: 10.1038/s41598-025-25764-0 (PMC12586693; doi:10.1038/s41598-025-25764-0)
Supplement: Supplementary file 5 — Supplementary Material 5 [file 41598_2025_25764_MOESM5_ESM.pdf]

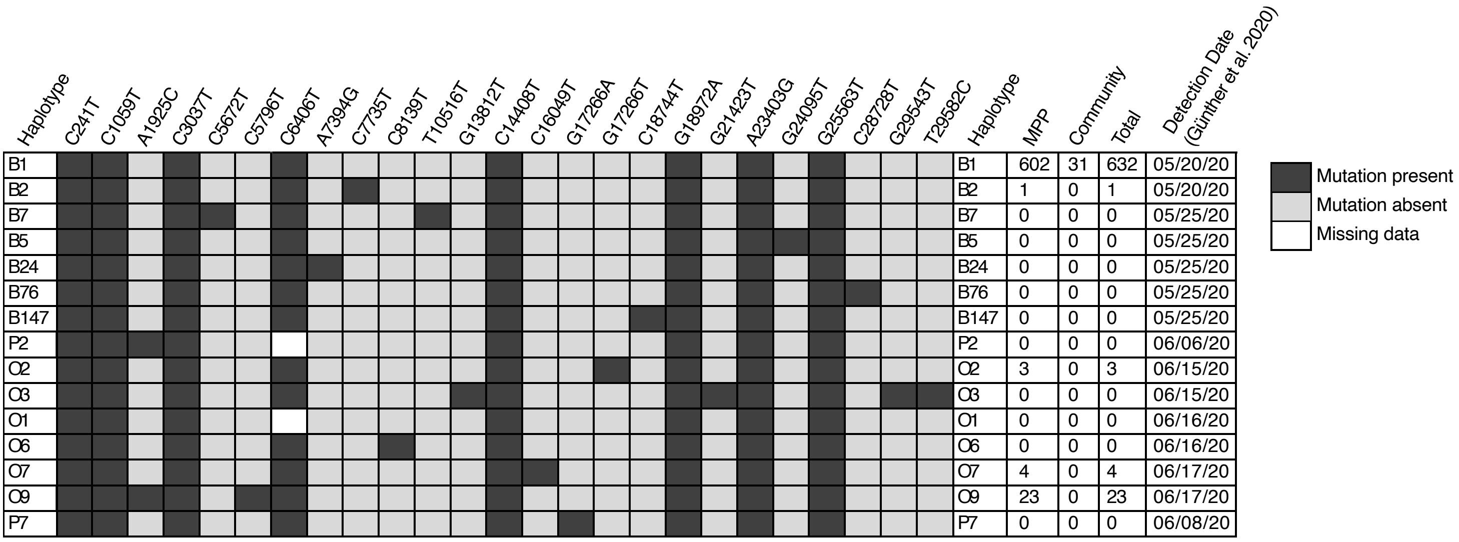

Supplementary Fig 1: Haplotypes from Günther et al. used in the sub-lineage analysis.

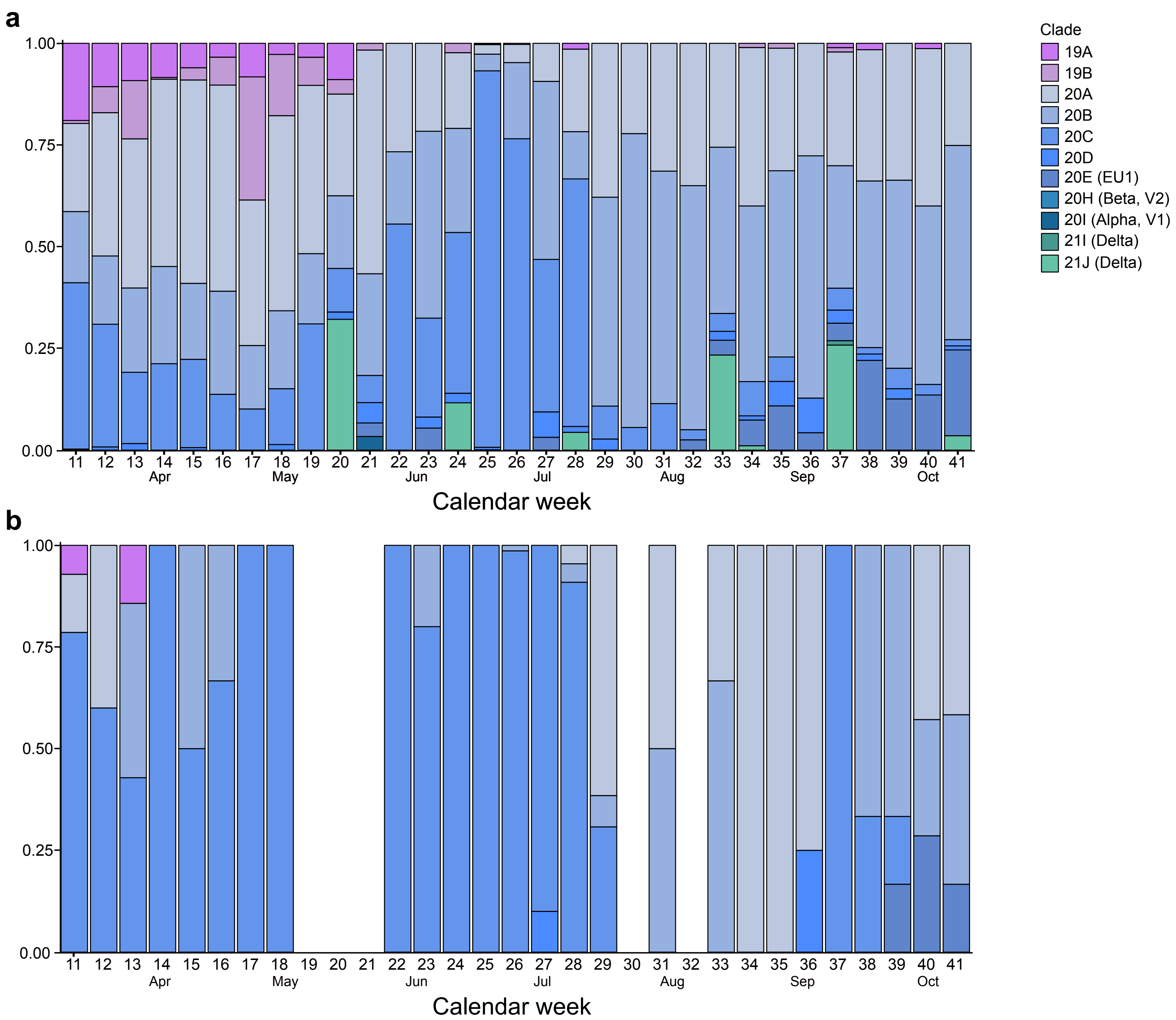

Supplementary Fig 2: Weekly clade distribution March-October 2020 in a) Germany and b) Gütersloh district (including the MPP).

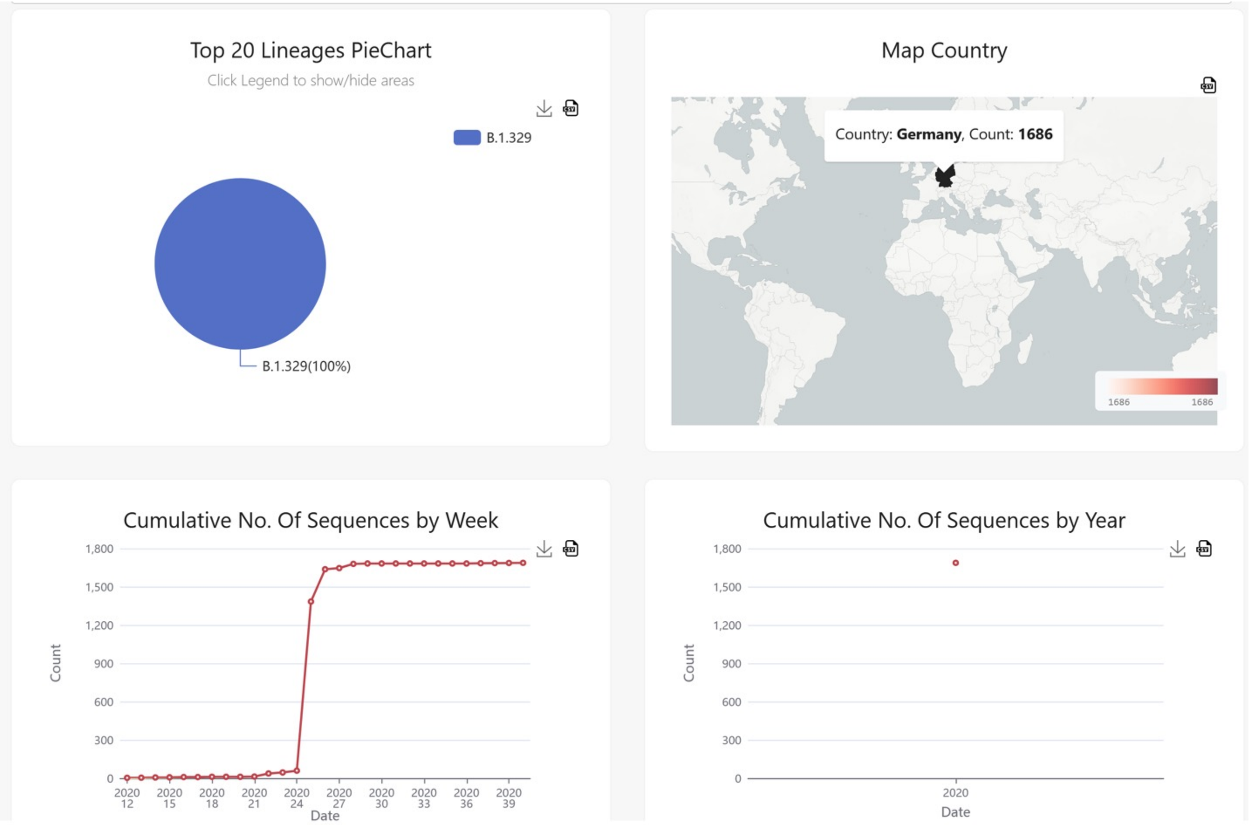

Supplementary Fig 3: GISAID results for B.1.329.

Supplementary File 1 (separate file). Analyzed consensus sequences.

Supplementary Table 1 (separate file). Weekly occurrence of Pango lineages for the community samples.

Supplementary Table 2 (separate file). Collection dates and locations for B.1.329-carriyng GISAID samples.

Supplementary Table 3 (separate file). Internal IDs to GISAID Accessions.
